# Supplementary material for: A network dynamical simulation model for the study of antibiotic resistance in microbial communities
Source: Gut Microbes. 2026 Sep 19;18(1):2734703. doi: 10.1080/19490976.2026.2734703 (PMC13596919; doi:10.1080/19490976.2026.2734703)
Supplement: Supplementary file 1 [file KGMI_A_2734703_SM9053.zip › supplementary_file_1/Figures/ba_delta_clustering.pdf]

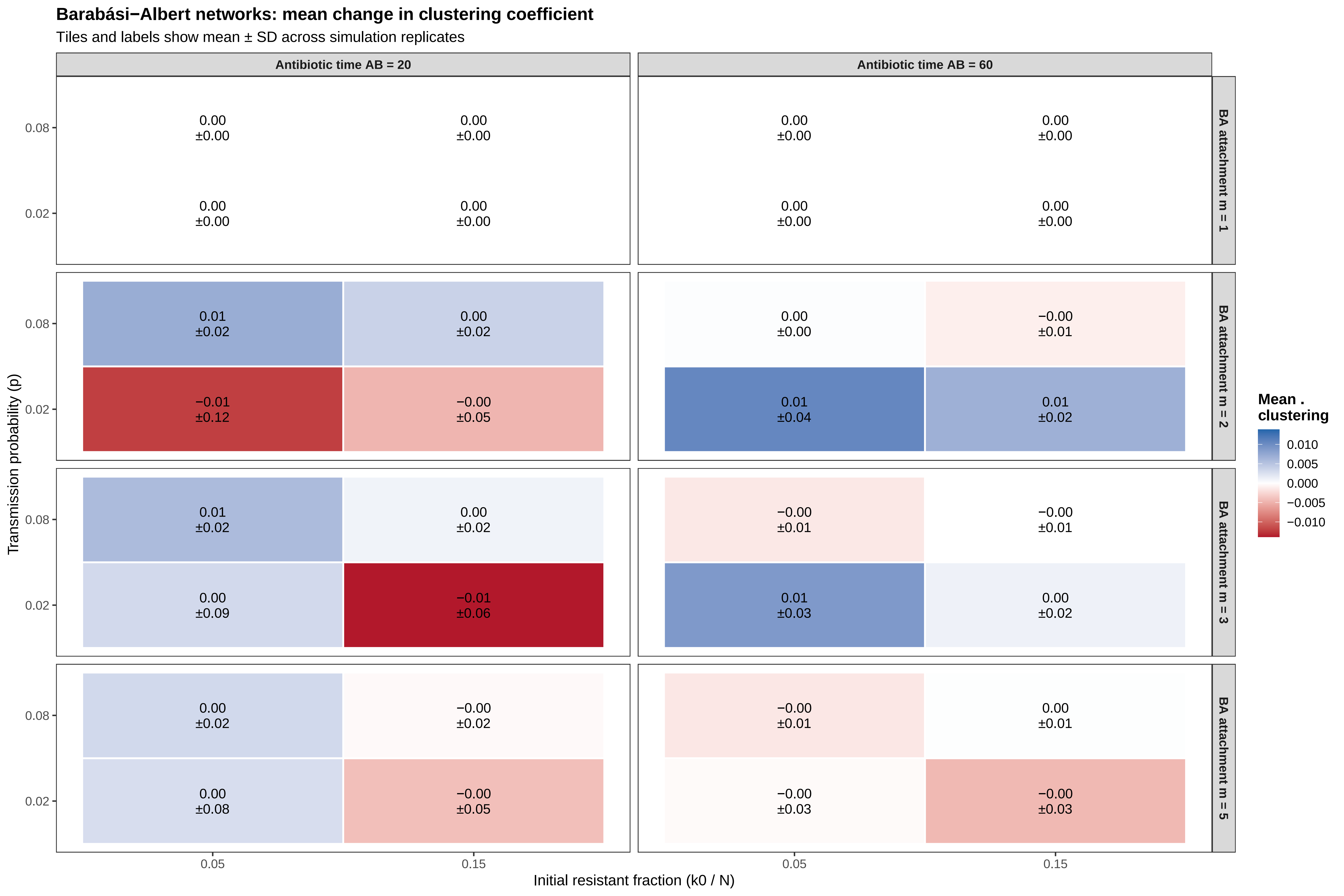

Barabási−Albert networks: mean change in clustering coefficient
Tiles and labels show mean ± SD across simulation replicates
Antibiotic time AB = 20
Antibiotic time AB = 60
BA attachment m = 1
BA attachment m = 2
BA attachment m = 3
BA attachment m = 5
Transmission probability (p)
Initial resistant fraction (k0 / N)
Mean . clustering
0.010
0.005
0.000
−0.005
−0.010
0.08
0.02
0.05
0.15
0.00 ±0.00
0.01 ±0.02
0.00 ±0.02
−0.00 ±0.01
−0.01 ±0.12
−0.00 ±0.05
0.01 ±0.04
0.00 ±0.09
−0.01 ±0.06
0.01 ±0.03
0.00 ±0.01
0.00 ±0.08
−0.00 ±0.02
−0.00 ±0.03
